# A Factorial Validation of Parental Mediation Strategies with Regard to Internet Use

**DOI:** 10.5334/pb.372

**Published:** 2017-06-26

**Authors:** Katrien Symons, Koen Ponnet, Kathleen Emmery, Michel Walrave, Wannes Heirman

**Affiliations:** 1 Higher Institute for Family Sciences, Odisee, Huart Hamoirlaan 136, 1030 Brussels, BE; 2 Department of Communication Studies, Media, ICT/Interpersonal Relations in Organizations and Society (MIOS), University of Antwerp, Sint-Jacobsstraat 2, 2000 Antwerp, BE; 3 Department of Communication Studies, Research Group for Media and ICT (MICT), Ghent University, Korte Meer 11, 9000 Ghent, BE; 4 Antwerp Maritime Academy, Noordkasteel Oost 6, 2030 Antwerp, BE

**Keywords:** Parental mediation, internet use, adolescents, multi-actor

## Abstract

This study investigated the strategies which parents employ in order to mediate their adolescent child’s internet use, thereby including the perspectives from the mother, the father and an adolescent child aged 13 to 18. Data from 357 families (*n* = 1071) were analyzed. Parental mediation strategies were inductively derived from a wide range of concrete mediation practices. Factor analysis yielded the same six factor solution for each informant, resulting in the identification of six distinct parental mediation strategies. Differences occurred between the three informants in terms of the quantity of mediation taking place. Parental mediation was predicted by the child’s age, but less by the parents’ age and the child’s gender.

The increased use of the Internet among young people has stimulated research on what parents can do to manage their children’s Internet use, in particular to prevent adverse outcomes associated with it (Duerager & Livingstone, 2012). Parental mediation is not specific to Internet use, however, and it has been described as referring to “the parental management of the relation between children and the media” (Livingstone & Helsper, 2008, p. 581). As discussed further in depth below, parental mediation research has particularly developed in the field of television viewing, and insights in this area have served as a theoretical basis for studying parental mediation of Internet use. However, in contrast to parental mediation of television viewing, no empirical consensus has been achieved with regard to the strategies that apply to parental mediation of Internet use (Livingstone & Helsper, 2008; Nikken & Jansz, 2013; Sonck, Nikken, & de Haan, 2013). Furthermore, over the past decade, Internet use among young people has continued to evolve in terms of access, usage, and risks (Hasebrink, 2014; Madden et al., 2013; Pew Research Center, 2016). It can be expected that parents have adapted their Internet parenting practices accordingly, so new research into parental mediation strategies that apply to Internet use is warranted.

The present study contributes to the empirical understanding of how parents currently mediate their children’s Internet use. Concretely, parental mediation strategies are distinguished in an inductive manner, departing from a wide range of parental mediation practices. A multi-actor approach is applied, taking into account reports from adolescents between the ages of 13 and 18, as well as both their mother and father. As discussed in depth below, former studies on the identification of Internet mediation strategies tended to include only one parent, usually the mother. Applying a multi-actor approach allows for taking into account discrepancies between the reports from the different actors and therefore improves the robustness of the findings. Finally, attention goes to differences in parental mediation according to the children’s and parents’ demographic characteristics.

## Theoretical Background

### Identifying Parental Mediation Strategies

Studies on parental mediation of children’s Internet use have heavily drawn from the well-established literature on parental mediation of television viewing. Thereby, parental mediation is defined as a “higher order construct” (Nathanson, 2001a, p. 119) considering the wide range of specific behaviors it may refer to. These behaviors can be grouped under a limited number of dimensions, however, commonly referred to as parental mediation styles or parental mediation strategies (e.g., Valkenburg, Krcmar, Peeters, & Marseille, 1999). To achieve an unambiguous identification of the styles or strategies that make up parental mediation, an inductive research approach is required whereby a wide range of possible mediation behaviors are grouped into a smaller number of internally consistent categories. Following such an approach, Valkenburg, Krcmar, Peeters, and Marseille (1999) achieved a classification of parental mediation styles in the area of television viewing. Their classification was based on parents’ reports on their engagement in a range of 30 mediation practices with reference to the television viewing of a child aged 5 to 12. By means of factor analysis, three factors were identified, which covered the following mediation styles: active mediation, referring to talking with children about television; restrictive mediation, referring to setting rules about children’s television viewing (how much, when, and which types of television can be viewed); and coviewing, referring to watching television with the child regardless of any communication taking place. The three-dimensional conceptualization of parental mediation of television viewing is widely accepted and applied (Nathanson, 2001b; Schofield Clark, 2011).

In order to manage their children’s Internet use, parents may engage in practices that are comparable to the mediation of television viewing. In addition, new practices have emerged that are Internet-specific. As such, blocking and filtering software have become available, which parents can use to manage their child’s access to certain Internet content (Lenhart & Madden, 2007; Mitchell, Finkelhor, & Wolak, 2005). These practices have been grouped under a new mediation strategy labelled technical mediation (Eastin, Greenberg, & Hofschire, 2006). As technology advanced, the range of practices that fall under this type of mediation extended to include, for example, the use of software to monitor online behavior or to limit Internet access (Nikken & Jansz, 2013). Another type of Internet-specific practices that became included in parental mediation research are practices to monitor the child’s activities after use, for example, by looking into the pages that were visited or by reading personal messages (Lenhart & Madden, 2007; Liau, Khoo, & Hwa Ang, 2008). Parallel to the literature on parental mediation of television viewing, variations exist in the way in which parental mediation of Internet use is conceptualized. Studies differ in the kind of mediation strategies that are included, as well as in the specific practices that are used to measure these strategies. In order to come to an unambiguous understanding of the parental mediation strategies that apply to Internet use, an inductive research approach is required. To the best of our knowledge, three such studies have investigated how a variety of parenting practices are grouped into aggregated parental mediation strategies.

The first study was conducted by Livingstone and Helsper (2008) and made use of data gathered among 9- to 17-year-olds, as well as one of their parents. The identification of parental mediation strategies was based on the parents’ reports on their engagement in 24 mediation practices. Factor analysis yielded a four-factor solution, distinguishing the following strategies: active co-use, interaction restrictions, technical restrictions, and monitoring. Active co-use refers to practices that cover both active mediation and co-use. Thus, in contrast to what was found for television viewing, these types of practices did not load on separate factors. The authors suggest that co-using the Internet by definition implies discussing the content, and therefore, both strategies become indistinguishable. Active co-use was the most popular parental mediation strategy. Interaction restrictions refer to rules regarding online interactions, such as using instant messaging and e-mail. Important limitations to the Livingstone and Helsper study were that some items cross-loaded on different factors, and not all the items theoretically fit the factors they were loading. Therefore, the four identified strategies were not entirely consistent.

The second study was conducted by Sonck, Nikken and de Haan (2013) and made use of data that were gathered among children aged 9 to 16, as well as one of their parents. Both the child and the parent were asked to indicate for the same 25 mediation practices whether these applied to them. The authors argued that, in order to be identified as a separate mediation strategy, it should come out as a factor for both the parent report and the child report. Following this approach, four mediation strategies were identified based on factor analysis. Two of these, monitoring and technical mediation, overlap with the strategies identified by Livingstone and Helsper (2008). A third strategy, restrictive content mediation, refers to restrictions in relation to all sorts of online activities, including using a social network profile, watching video clips online, using instant messaging, and downloading music or films. Giving out personal information and uploading media content did not, however, load on this factor in a consistent manner. Thus, the restrictions that came out as a separate factor in this study are distinct from what were identified as interaction restrictions by Livingstone and Helsper (2008). The fourth factor that was identified was active safety mediation, labelled as such because it refers to parents discussing practices to enhance online safety. Unlike what was found by Livingstone and Helsper, active co-use could not be identified as a distinct strategy. With regard to these items (e.g., talking about what the child does online, or doing shared activities online), children and their parents held incongruent perspectives, leading the authors to conclude that active co-use did not represent a distinct strategy.

The third study was conducted by Nikken and Jansz (2013), who made use of data gathered from one parent of a child aged 2 to 12. Factor analysis on 20 mediation items yielded a five-factor solution, referring to clearly distinct mediation strategies. The first three strategies—active mediation, co-use, and restrictive mediation—overlap with the three traditional mediation strategies as identified in the field of television viewing by Valkenburg et al. (1999). This overlap could be related to the fact that both studies used reports from parents with a young child, whereas the two studies discussed above were based on practices with adolescents. Restrictive mediation was divided into two subtypes, namely restrictions on general access (when/how long the child can use the Internet, and suitability of certain games) and restrictions on specific content (e.g., what can be downloaded or bought online), which is different from what was found by Valkenburg et al. (1999). A fifth and entirely new strategy was supervision, referring to being around or nearby when the child is online. Supervision was also the most popular type of mediation. Items referring to monitoring did not load on a separate factor, implying that in this age category, monitoring is not a separate mediation strategy. The study did not identify interaction restrictions as a separate strategy, which could be due to the absence of applicable items included in the factor analysis. Interaction restriction practices might also be less relevant for younger children because they access social network sites to a lesser extent compared to older children (Houghton et al., 2015). Items referring to technical mediation were not included in the factor analysis.

To summarize, the studies cited above demonstrate that the traditional, three-dimensional understanding of parental mediation is inaccurate with regard to Internet use. They did not, however, come to a definite conclusion with regard to the strategies that can be distinguished. The studies support the identification of technical mediation as a stand-alone strategy. Also, active mediation and restrictive mediation emerged as separate strategies across the different studies, albeit in different versions. Monitoring only came out as a distinct strategy in the older age group, while co-use and supervision only came out as separate strategies in the younger age group. A shortcoming of these studies is that they did not include the perspectives of all family members involved. As discussed in the next section, there is a need to identify both parents’ perspectives as well as the child’s in order to come to a full understanding of parental mediation.

### Including Different Actors’ Perspectives

It can be argued that the identification of mediation strategies from a multi-actor approach yields more robust results compared to studies that only include the perspective of one actor. Particularly, discrepancies between parents and their children in reports on mediation have been established in the literature. For example, a study on parental mediation practices with regard to overall media use found that parents are more inclined than children to indicate that certain rules apply (Livingstone, 2007). Parent-child discrepancies have also been found with regard to rules concerning specific Internet activities (Livingstone & Bober, 2004; Wang, Bianchi, & Raley, 2005) and with regard to parental monitoring activities, supervision, and communication (Liau et al., 2008). There are indications, however, that the parent-child discrepancies mostly refer to the amount of mediation taking place rather than to the types of mediation that apply to Internet use. In the study by Sonck et al. (2013), the same four mediation strategies were identified based on the child report and parent report, and inconsistent factor loadings between groups only emerged for a select set of items. Furthermore, they found that children consistently reported less mediation as compared to their parents, but both agreed that active mediation was the most commonly applied strategy, followed by monitoring. Also, a study on parental mediation of video gaming came to similar conclusions. Children consistently reported less mediation than their parents, while in relative terms, children and parents did agree on which mediation strategy was most commonly applied (namely restrictive mediation; Nikken & Jansz, 2006). When it comes to reports on the amount of mediation, girls typically report receiving more mediation as compared to boys, although research results are not entirely consistent (Álvarez, Torres, Rodríguez, Padilla, & Rodrigo, 2013; Livingstone, Kalmus, & Talves, 2014; Wang et al., 2005). The clearest predictor of parental mediation on the level of the child is the child’s age. Parental mediation declines as the child grows older (Álvarez et al., 2013). More specifically, parents decrease their monitoring and restrictive mediation as the child gets older (Padilla-Walker, Coyne, Fraser, Dyer, & Yorgason, 2012; Sonck et al., 2013).

So far, no study has been conducted in which the identification of parental mediation strategies is based on the perspective of both parents. It is plausible, however, that strategies identified based on mothers’ reports will yield different results as compared to findings based on fathers’ reports. Research suggests that—in Western countries—fathers are less involved in parenting than mothers. While fathers’ involvement has increased over the second half of the 20th century (Bianchi, 2000), mothers still spend more time with their children and take on more responsibilities in the child-rearing compared to fathers (Phares, Fields, & Kamboukos, 2009). Also, when it comes to parental mediation of children’s Internet use, mothers are more engaged than fathers. For example, the Internet parenting style of mothers shows more elements of parental control (referring, e.g., to supervision and setting restrictions) as well as parental warmth (referring to support and communication) compared to fathers (Valcke, Bonte, De Wever, & Rots, 2010). A study in the UK with regard to the regulation of media use in general found that children perceive mothers to be more restrictive than fathers. Nevertheless, mothers’ and fathers’ accounts of regulatory practices were similar, apart from mothers being more likely to restrict the child’s use of the telephone (Livingstone, 2007).

## The present Study

Based on the literature, three research goals were formulated. The first goal was to identify the parental mediation strategies in the field of Internet use by testing how a range of parental mediation practices as reported by the mother, the father, and the child group onto higher order mediation strategies. The second goal was to understand discrepancies in the amount of mediation taking place as reported by both the parents and the child. The third goal was to investigate the extent to which parents’ age and gender, as well as the child’s age and gender, are predictive for parental mediation. The study focuses on parental mediation of adolescents aged 13 to 18.

## Method

### Participants and Procedure

A multi-actor approach of data collection was used, following the procedure outlined in the relationship between mothers, fathers, and children (RMFC) study (Ponnet, 2014; Ponnet & Wouters, 2014). Two-parent families were recruited in order to obtain a report from the mother, the father, and a child in the age group of 13 to 18 years old. In the case of newly composed families, it was requested that both partners had shared the same house for at least three years prior to the survey. This was done in order to ensure that the bond between the non-biological parent or caregiver and the child was sufficiently established. If there was more than one child in the family between 13 and 18 years old, the parents were asked to keep one specific child in mind when completing the questionnaire. Given the high rate of non-response associated with the collection of multi-actor data (Kalmijn & Liefbroer, 2011), the study employed a non-probabilistic sampling design.

Families were recruited with assistance from undergraduate students from the higher education institution where the researchers are based. Each recruited family received an envelope containing the three questionnaires for the participating family members, along with a plain-language statement and a written informed-consent form. The first page of the questionnaire instructed the target participants to complete the booklets individually and not to discuss the content of the questionnaire with one another. In order to protect the respondents’ privacy, separate envelopes were provided that could be sealed and used for each completed questionnaire. After completion, the three questionnaires were sent back by regular mail, for which a (stamped) envelope was provided. Data were gathered between December 2015 and February 2016. In total, 365 triads were achieved, of which 357 were retained after data cleaning. The study protocol was approved by the ethics committee of Antwerp University.

### Measures

**Demographic characteristics.** The children’s ages ranged from 13 to 18 (*M* = 15.73; *SD* = 1.50), the mothers’ ages ranged from 31 to 59 (*M* = 44.19; *SD* = 4.72), and the fathers’ ages ranged from 31 to 70 (*M* = 46.67; *SD* = 5.65). For children, gender was also included (54.9% female).

**Parental mediation practices.** The parent and child questionnaires contained the same set of questions referring to parental mediation practices. A list of 19 practices covering active and restrictive mediation practices was used. The items were largely derived and adapted from former studies, mainly the studies by Livingstone and Helsper (2008) and Sonck et al. (2013). Most items were measured on the binary level, with the categories of “yes” (the practice or rule applies) versus “no” (the practice or rule does not apply). For some items, additional answering options were included. For example, for the items referring to rule-setting, distinction was made between “the rule does not apply,” “the rule applies in a flexible way,” and “the rule applies in a strict way.” For reasons of consistency, these items were dichotomized by merging the last two categories. Some items were measured in terms of the frequency with which the parents engaged in it. These items were answered on a five-point Likert scale ranging from 1 (*[almost] never*) to 5 (*always*). For an overview of the items and the descriptors, refer to Appendix 1.

### Analyses

The first research goal (i.e., identifying the parental mediation strategies with regard to Internet use) was achieved by applying factor analyses based on the 19 mediation practices. Analyses were done separately for the children, mothers, and fathers. As the extraction method, principal component analysis was used. Because it was likely that the extracted factors would correlate with each other, the oblique rotation option was selected. Extraction was based on achieving an Eigenvalue greater than 1. To deal with missing values, pairwise deletion was selected. Informed by the results of the factor analyses, an aggregated variable was constructed for each identified parental mediation strategy. The second research goal (i.e., unravelling discrepancies on the amount of mediation taking place as reported by both parents and the child) was achieved by applying repeated measures ANOVA. This method was appropriate as the mediation variables are mutually related to each other. The difference between the three variables is merely the actor from whose perspective the items were completed. To achieve the third research goal, a series of linear regression analyses was applied in order to test for the relative importance of the parents’ and the child’s age as well as the child’s gender on the amount of mediation as reported by each actor. SPSS Statistics 22 was used.

## Results

### Research Goal 1: The Identification of Parental Mediation Strategies

An examination of the Kaiser-Meyer Olkin measures of sampling adequacy showed that the samples derived from the three respective informants—the adolescent, the mother, and the father—were each suitable for factor analysis (KMO_adolescent_ = .752; KMO_mother_ = .789; KMO_father_ = .815, each with *p* < .001). Two items were excluded from the analyses because they did not load on separate factors in a consistent manner, namely the items “being friends with a parent/with the child on the social network” and “parents have access to the login credentials of the child’s social network profile(s) and email.” In the child group, these items loaded on a separate factor, while in both the parents’ groups, these items loaded on a factor together with other monitoring practices. In addition, the item “having access to the login credentials” loaded on two different factors for both the child and the father sample. Therefore, the factor analyses were repeated with exclusion of these two items. For each sample, the principal component analysis revealed a six-factor solution, with a total explained variance of 63.77% for the children, 68.02% for the mothers, and 71.43% for the fathers. Tables 1, 2 and 3 show, for each respective group, the factor loadings for the 17 items that were included in the analyses. The loadings from the pattern matrix are displayed, which show the unique relationship between the factor and each indicator, while controlling for the variance that is explained by the other factors (Brown, 2015, p. 28). The tables show that for each sample, the exact same six factors were found, albeit in a slightly different order of relevance. Also, the factor loadings for the individual items were similar across the different groups.

**Table 1:** Results of the principal component analysis based on the children’s reports.

	Interaction restrictions	Monitoring SN use	Access restrictions	Superv./co-use	Technical mediation	Interpret. mediation

Parents log in to profile to read messages	.051	**.820**	–.029	–.084	–.020	–.012
Parents check SN page	–.026	**.654**	.080	.168	–.005	–.021
Parents check contacts on SN	–.004	**.879**	.013	–.050	.034	.062
Parents watch when using the I	.019	.228	.073	**.673**	.034	–.052
Parents help when using the I	.064	.038	–.273	**.694**	.116	.147
Parents are around when using the I	.018	–.170	.180	**.745**	–.096	–.045
Software to limit I access (time)	.092	–.029	–.049	–.040	**.836**	–.125
Parents use software to block websites	–.117	.025	.118	.049	**.808**	.112
Discussed not everything online is true	.046	–.027	.014	–.015	.018	**.832**
Discussed potential dangers of the I	.003	.045	.057	.011	–.047	**.807**
Rules – the time you can spend online	.217	.107	**.608**	–.024	.097	–.026
Rules – times of the day you can be online	–.001	–.113	**.698**	.014	.037	.143
Rules – the use of the I in the bedroom	–.011	.134	**.784**	.031	–.020	–.010
Rules – the pictures you can post	**.789**	–.033	.050	.017	.044	–.028
Rules – the information you can share	**.731**	–.022	.097	.064	.075	.019
Rules – with whom you can chat	**.781**	.009	.031	.018	–.100	–.011
Rules – who you can add to SN	**.827**	.050	–.103	–.042	–.012	.078

Explained variance (R^2^)	22.95%	10.55%	8.47%	7.63%	7.43%	6.73%

SN = social network; I = internet; Superv. = supervision; Interpret. = interpretative.

**Table 2:** Results of the principal component analysis based on the mothers’ reports.

	Interaction restrictions	Monitoring SN use	Access restrictions	Technical mediation	Superv./co-use	Interpr. mediation

Logging in to child’s profile to read messages	–.035	**.878**	–.007	–.011	–.067	–.068
Checking child’s SN page	.020	**.755**	.092	–.005	.079	.074
Checking added contacts to child’s SN	.072	**.871**	.021	–.005	.041	.024
Watching when the child uses the I	.062	.016	.045	.032	**.809**	–.014
Helping the child using the I	–.061	.111	–.096	.017	**.783**	.016
Being around when the child uses the I	.080	–.113	.222	–.086	**.730**	–.002
Software to limit I access in time	–.067	–.060	.155	**.767**	–.043	.048
Software to block access to certain websites	.116	.042	–.077	**.799**	.022	–.039
Discussed that not everything online is true	–.041	.016	–.089	.104	.124	**.690**
Discussed the potential dangers of the I	.054	–.008	.075	–.094	–.126	**.809**
Rules – the time the child can spend online	–.020	–.006	**.743**	.066	.115	.013
Rules – times of the day the child can be online	–.043	.105	**.822**	.058	–.043	–.028
Rules – the use of the I in the bedroom	.140	.017	**.764**	–.039	.046	.010
Rules – the pictures the child can post online	**.792**	–0.31	.154	–.045	–.033	.003
Rules – the information the child can share	**.820**	–.012	.024	–.023	.011	.010
Rules – with whom the child can chat	**.863**	.019	–.082	.100	.014	–.012
Rules – who the child can add to SN	**.834**	.072	–.065	.014	.034	.016

Explained variance (R^2^)	27.23%	10.52%	9.65%	7.36%	7.13%	6.13%

SN = social network; I = internet; Superv. = supervision; Interpret. = interpretative.

**Table 3:** Results of the principal component analysis based on the fathers’ reports.

	Interaction restrictions	Monitoring SN use	Access restrictions	Interpret. mediation	Technical mediation	Superv./co-use

Logging in to child’s profile to read messages	.071	**.789**	.016	–.088	.164	–.010
Checking child’s SN page	–.057	**.857**	–.012	.114	–.153	.030
Checking added contacts to child’s SN	.105	**.855**	.066	–.083	.032	.014
Watching when the child uses the I	.047	–.016	.082	.002	.079	**.815**
Helping the child using the I	–.068	.195	–.076	.194	.049	**.639**
Being around when the child uses the I	.115	–.071	.168	–.070	–.116	**.723**
Software to limit I access in time	–.033	.040	.147	.109	**.741**	–.195
Software to block access to certain websites	.028	–.006	–.109	–.073	**.808**	.207
Discussed that not everything online is true	.062	–.003	.072	**.839**	–.033	–.012
Discussed the potential dangers of the I	.038	–.038	–.037	**.861**	.056	.061
Rules – the time the child can spend online	.005	–.030	**.804**	.020	.127	.044
Rules – times of the day the child can be online	–.074	.034	**.865**	.029	–.107	.013
Rules – the use of the I in the bedroom	.124	.057	**.730**	–.022	.022	.063
Rules – the pictures the child can post online	**.822**	.063	–.012	.072	.000	–.034
Rules – the information the child can share	**.798**	.053	–.021	.095	–.047	.032
Rules – with whom the child can chat	**.868**	–.009	.036	–.040	.033	.013
Rules – who the child can add to SN	**.910**	–.033	–.006	–.025	–.006	.004

Explained variance (R^2^)	30.45%	10.06%	9.07%	8.23%	7.04%	6.58%

SN = social network; I = internet; Superv. = supervision; Interpret. = interpretati.

The first factor is labeled “interaction restrictions” and refers to rules and restrictions related to activities on the social network. For both the parents and the adolescent, this was by far the most important strategy. The second factor is labeled “monitoring” and refers to practices to gather knowledge about the child’s behavior on the social network. The third factor, “access restrictions,” refers to rules and restrictions related to Internet access, namely when, for how long, and where the child can be online. The fourth factor refers to “supervision and co-use,” with both types of practices loading on the same factor. The fifth factor, labeled “technical mediation,” refers to the application of software for limiting Internet access or for blocking access to certain websites. The final factor refers to parents discussing Internet content and is labeled “interpretative mediation.”

### Research Goal 2: Investigating Discrepancies in Reported Amount of Mediation

For each identified mediation strategy, one variable was constructed by calculating the aggregated mean value, including all respective items. Table 4 shows the univariate results for each constructed variable, together with the Cronbach’s alpha, indicating the internal consistency for the respective items. When working with dichotomous items only (as is the case for the aggregated variables interaction restrictions, access restrictions, technical mediation, and interpretative mediation), SPSS automatically offers the KR20 measure for internal consistency, which is more appropriate when working with dichotomous items (Sijtsma, 2009). The results show a low internal consistency score for the items measuring technical mediation for the child’s and both parents’ reports. Internal consistency was also low for the items measuring interpretative mediation for the mother, as well as the items measuring supervision/co-use for the child.

**Table 4:** Parental mediation strategies as perceived by the child, the mother and the father, with parent-child paired samples t-test.

	Child			Mother			Father			

	Range (*M*)	*SD*	α	Range (*M*)	*SD*	α	Range (*M*)	*SD*	α	F

Interaction restr.	0–1(.39) (i)	.38	.808	0–1(.58) (i)	.41	.859	0–1(.48) (i)	.43	.887	32.73***
Monitoring SN use	1–5(1.71) (i)	.85	.718	1–5(2.19) (i, ii)	.93	.812	1–4.67(1.74) (ii)	.83	.809	34.37***
Access restrictions	0–1(.47) (i)	.37	.626	0–1(.59) (i)	.39	.756	0–1(.53) (i)	.41	.773	15.62***
Technical mediation	0–1(.08)	.24	.482	0–1(.11)	.25	.413	0–1(.10)	.24	.402	1.44*
Interpret. mediation	0–1(.87) (i)	.29	.594	0–1(.97) (i, ii)	.14	.243	0–1(.90) (ii)	.26	.702	20.82***
Supervision/co-use	1–4.33(2.40) (i)	.75	.538	1–5(2.78) (i, ii)	.84	.730	1–4.33(2.42) (ii)	.80	.679	28.15***

Note: α = Cronbach’s alpha/KR20; restr. = restrictions; Interpret. = interpretative; Level of significance for the difference between the child and each respective parent, tested by repeated measures ANOVA, Wilk’s Lambda **p < .01, ***p < .001; (i) and (ii) = outcome for the post-hoc pairwise comparison (Bonferroni), different actors’ outcomes differ significantly.

Table 4 also includes the F-values and significance levels for the Wilks’ Lambda multivariate test, indicating the statistical significance of different outcomes between the actors. The results show that the child’s, the mother’s, and the father’s reports differed significantly for each type of mediation. Post-hoc pairwise comparisons (i.e., Bonferroni tests) were applied in order to disentangle which specific actors differ from each other. Concerning restrictions on the behavior that is allowed on social networks and restrictions on access to the Internet in general, children perceive significantly less mediation than what is reported by the mother and the father. For these factors, mothers also reported significantly more mediation than fathers. With regard to monitoring practices, interpretative mediation, and supervision/co-use, children reported significantly less mediation than the mother, but not less than the father. For these types of mediation, mothers scored significantly higher than fathers. For technical mediation, no differences were found among the different actors’ reports.

### Research Goal 3: Investigating the Effect of Age and Gender on Parental Mediation

Table 5 shows the results for the linear regression analyses, measuring the effects of age and gender on parental mediation as reported by the child, the mother, and the father. Because the ages of the child and both parents correlated with each other (*r*(351) = .29, *p* < .001 for child’s age and mother’s age; *r*(339) = .27, *p* < .001 for child’s age and father’s age; and *r*(334) = .67, *p* < .001 for mother’s age and father’s age), a test for multicollinearity was performed for these independent variables. The results indicated that multicollinearity was not a concern, applying a VIF threshold of 2.5 (age child, VIF = 1.12; age mother, VIF = 1.88; and age father, VIF = 1.83). The table shows that for each group, the most consistent and strongest predictor for parental mediation is the child’s age. From the fathers’ perspective, the child’s age is the only factor that is significantly related to mediation. As expected, children receive less mediation as they grow older, and the effect of age is most pronounced when it concerns interaction and access restrictions. Remarkably, from the perspective of the child, age did not have a significant effect on supervision and co-use, but both parents indicated engaging less in this type of mediation with increasing age of the child. There is only limited evidence for gendered socialization in the field of Internet use. Compared to boys, girls perceive significantly more interaction restrictions. Also, mothers indicated engaging more in this type of mediation with girls, but for fathers, there is no such effect. When it comes to Internet access in general, mothers were found to impose fewer restrictions on girls as compared to boys. Only a few significant effects were found for parents’ age. Specifically, children perceived more technical mediation when their mother was younger, and more monitoring when their father was younger. Mothers reported less monitoring and supervision/co-use when they were older. From the father’s perspective, age did not have any effect on engagement in parental mediation.

**Table 5:** Predictors of parental mediation according to the child, the mother, and the father (Standardized beta-scores).

	Interaction restrictions	Monitoring SN use	Interpret. mediation	Access restrictions	Technical mediation	Superv./co-use

Child report						
Gender child (ref. = boy)	0.145**	–0.043	0.048	0.008	0.002	0.026
Age child	–0.329***	–0.188**	–0.212***	–0.360***	–0.081	–0.098
Age mother	–0.121	0.007	–0.031	–0.059	–0.221**	–0.108
Age father	–0.004	–0.188*	–0.069	0.006	0.115	0.062
Adj. R²	.146	.079	0.053	.133	.027	.007
Mother’s report						
Gender child (ref. = boy)	0.135*	0.077	–0.006	–0.105*	0.050	0.016
Age child	–0.356***	–0.289***	–0.178**	–0.406***	–0.037	–0.288***
Age mother	–0.062	–0.170**	0.084	–0.019	–0.076	–0.199***
Adj. R²	.143	.132	.022	.182	.002	.147
Father’s report						
Gender child (ref. = boy)	0.056	–0.026	0.077	–0.084	0.025	–0.015
Age child	–0.337***	–0.274***	–0.161**	–0.401***	–0.102	–0.308***
Age father	–0.063	–0.093	–0.036	–0.022	0.064	–0.091
Adj. R²	.119	.090	.025	.172	.002	.111

SN = social network; Interpret. = interpretive; Superv. = supervision; *p < .05; **p < .01; ***p < .001.

## Discussion

The present study identified parental mediation strategies in the field of adolescents’ Internet use, taking into account the mother’s, father’s, and adolescent’s perspective. The inclusion of these three informants fills an important gap in the research literature. To date, research on parental mediation tended to include the perspective of only one parent, usually the mother. Fathers, however, are currently more involved in rearing their children than was the case in the past. Although most fathers do not take as active a role in the parenting process as most mothers do, the gap between men’s and women’s participation in child-rearing appears to be shrinking (Amato, Meyers, & Emery, 2009; Ponnet, Van Leeuwen, Wouters, & Mortelmans, 2015). Building on former studies, a wide range of parenting practices were included and grouped under distinct strategies by applying principal component analysis. Furthermore, the study investigated differences between the informants in terms of the amount of mediation that is reported, as well as the effect of the parent’s and child’s age and gender on parental mediation.

The principal component analysis resulted in a six-factor solution, which was consistent across the different actors. The six strategies that were identified overlap with what was found in former studies in which mediation strategies were inductively distinguished, namely the studies by Livingstone and Helsper (2008) and Sonck et al. (2013) with adolescents, and the study by Nikken and Jansz (2013) with younger children. First, interaction restrictions refer to rules about appropriate behavior on social network sites. This strategy was also found by Livingstone and Helsper (2008) and Sonck et al. (2013). Second, monitoring refers to the ad-hoc checking of the child’s online behavior, which is also in line with the findings by Livingstone and Helsper (2008) and Sonck et al. (2013). Third, access restrictions refer to rules about when, for how long, and where the child can go online. In the Livingstone and Helsper’s (2008) study, only one item was included referring to access restrictions (“rules about the time spent online”), which loaded on a strategy labelled active co-use. In the Sonck et al. (2013) study, no items referring to access restrictions were included. Hence, no such strategy was distinguished. The study by Nikken and Jansz (2013) did distinguish a strategy referring to restrictions on general Internet access. This further supports the conclusion that access restrictions are a valid stand-alone strategy. Fourth, interpretative mediation refers to parents discussing Internet content. This overlaps with what was found by Nikken and Jansz (2013) with parents of younger children. In the Livingstone and Helsper (2008) study, interpretative mediation loaded together with items referring to co-use and was therefore labeled “active co-use.” In the present study, however, co-use was a distinct strategy that loaded together with supervision. In the Sonck et al. (2013) study, items referring to interpretative mediation loaded on one factor, which was labeled active safety mediation because it specifically included items referring to discussing online safety. Fifth, technical mediation was a distinct strategy, which confirms what was found by Livingstone and Helsper (2008) as well as Sonck et al. (2013). Nikken and Jansz (2013) did not include items referring to technical mediation in their factor analysis. While the present study confirms technical mediation as a distinct strategy, only a minority of respondents reported engaging in this. The sixth and final strategy that was identified was supervision/co-use. While in the Livingstone and Helsper (2008) study, co-use loaded together with items referring to interpretative mediation, it was a separate strategy in the present study. In the Sonck et al. (2013) study, these practices did not load on the same factor across the different informants (parent and child). Therefore, the authors concluded that this is not a separate strategy. In the Nikken and Jansz (2013) study, active co-use and supervision were identified as two distinct strategies. It is possible that the distinction between supervision and co-use as separate strategies is specific for younger children while both types of practices are more likely to coincide when it concerns older adolescents.

Discrepancies were found between the different informants in terms of the amount of mediation that takes place. In line with previous studies, children perceived less mediation as compared to their parents (Liau et al., 2008; Sonck et al., 2013; Wang et al., 2005). This is a general trend that is also found in the field of parental monitoring of offline behaviors, with children consistently reporting less monitoring as compared to the parents (Abar, Jackson, Colby, & Barnett, 2015). Children may not always be aware of their parents’ deliberate mediation or monitoring efforts. In addition, parents may be motivated to exaggerate their parenting activities while children may be motivated to exaggerate their personal autonomy. Differences were also found between parents, with fathers indicating less mediation as compared to mothers. This suggests that mothers’ increased involvement in the child’s upbringing as compared to the fathers’ also applies to an increased involvement in the child’s Internet use.

Parental mediation was largely predicted by the child’s age, but less by the child’s gender and the parents’ ages. In line with the expectations, all informants report less mediation as the child grows older. Research suggests that parents become increasingly permissive and laissez-faire in their Internet parenting as the child gets older (Özgür, 2016). A remarkable exception to this is that children do not perceive less supervision/co-use when they are older, but both parents do indicate engaging less in this type of mediation with older children. It is not clear how this finding can be explained. It should also be pointed out that parents do not necessarily become uninvolved in their older children’s Internet use. It is possible that parents will apply different strategies that are not typically included in parental mediation research. A study by Padilla-Walker et al. (2012) supports this suggestion. In the study, “deference” was included as a distinct strategy for managing the child’s Internet use. Deference implies that parents actively choose not to intervene and to grant autonomy to their child as long as the media influence does not negatively impact the child’s behaviors. Based on a longitudinal design, they found that restrictive and active monitoring decreased over time while deference increased.

The amount of parental mediation did not depend on the child’s gender, with a few exceptions. For instance, the child and the mother reported more interaction restrictions for girls than for boys, and mothers reported fewer access restrictions for girls than for boys. The higher level of interaction restrictions for girls could potentially be linked to increased social anxieties around girls’ vulnerability to contact risks such as grooming (Pedersen, 2013). In addition, research on sharing suggestive pictures and its potential negative effects on body image has largely focused on girls, while boys may be vulnerable to such effects as well (de Vries, Peter, de Graaf, & Nikken, 2016). Girls also spend more time on social networks as compared to boys (Tsitsika et al., 2014). Hence, they may be perceived as being more in need of interaction restrictions. Parents’ age did not play a significant role, which is different from previous studies suggesting that parents engage less in mediation when they are older (e.g., Álvarez et al., 2013). There were some exceptions for the mother’s age, with the child perceiving less technical mediation when the mother was older and mothers reporting less supervision/co-use when they were older.

This study contributes to the current body of research on what parents can do to enhance the safe Internet use of their children. Previous research has yielded inconclusive results as to the strategies that are most effective. Overall, it is suggested that active mediation strategies are more successful in the prevention of online risks as compared to restrictive mediation strategies (Ang, 2015). Nevertheless, the protective effect of restrictive practices has also been shown repeatedly (Lee, 2012; Navarro & Jasinski, 2012; Navarro, Serna, Martínez, & Ruiz-Oliva, 2013). The wide variety in the specific parenting practices that were included in such studies, however, renders the comparison of research results problematic. The research on parental mediation would benefit from more conceptual clarity around the topic. The results of the present study can add to such conceptual clarity by investigating the strategies that make up parental mediation. Furthermore, the findings suggest that a multi-actor approach is not required as much when it comes to identifying the strategies that apply, but that it is important to consider that different actors have different perceptions of the amount of mediation taking place.

Despite the strengths of this study, it is important to note some limitations. The first limitation is sampling bias, which may limit the generalizability of the study findings. Only two-parent families with at least one child between 13 and 18 years old were recruited, thereby excluding single parents. Alternative participant recruitment and data collection strategies might be needed to ensure whether the findings remain true in single-parent families. A second limitation is that the internal consistency of some of the constructed mediation variables was low. For technical mediation, a low consistency score was found for each informant; for interpretative mediation, a low score was found for the mother; and for supervision/co-use, a low score was found for the child. While there is no gold standard for how high a Cronbach’s alpha score should be for adequate reliability, a low score does raise concerns about internal consistency. An explanation for the low internal consistency scores was that only two items were used to measure technical mediation and interpretative mediation. Furthermore, in the case of technical mediation, there was only very limited variation because only a few parents engaged in this strategy. Future research should pay more attention to the measurement of these strategies. Finally, the study did not investigate differences in mediation practices according to parents’ ethnic and socioeconomic background. The sample that was used for this study was rather homogenous in terms of these background characteristics, and no measures were taken to recruit the harder-to-reach groups of parents. We therefore acknowledge that our participants come from a relatively privileged group in terms of the resources they have for performing parental mediation.

## Additional File

The additional file for this article can be found as follows:

10.5334/pb.372.s1 Appendix Description of parental mediation items from the perspective of the child, the mother and the father.
