# Supplementary material for: A Factorial Validation of Parental Mediation Strategies with Regard to Internet Use
Source: Psychol Belg. 2017 Jun 26;57(2):93–111. doi: 10.5334/pb.372 (PMC6194521; doi:10.5334/pb.372)
Supplement: Appendix — Description of parental mediation items from the perspective of the child, the mother and the father. [file pb-57-2-372-s1.pdf]

Appendix 1. Description of parental mediation items from the perspective of the child, the mother and the father

	Child<br>*M* (SD)	Mother<br>*M* (SD)	Father<br>*M* (SD)
How often… (scale 1 – 5)			
Logging in to child's profile to read messages	1.37 (.892)	1.78 (1.014)	1.50 (0.839)
Checking child's SN page	2.41 (1.196)	2.74 (1.198)	2.09 (1.129)
Checking added contacts to child's SN	1.43 (0.882)	2.04 (1.060)	1.63 (0.927)
Watching when the child uses the I	1.74 (0.867)	2.55 (1.006)	2.12 (0.948)
Helping the child using the I	2.14 (1.043)	2.54 (0.963)	2.41 (0.913)
Being around when the child uses the I	3.31 (1.173)	3.24 (1.151)	2.75 (1.186)
Engagement in or having rules about… (yes/no)	Child<br>% yes	Mother<br>% yes	Father<br>% yes
Software to limit I access in time	3.5%	6.7%	6.0%
Software to block access to certain websites	14.1%	14.1%	13.7%
Discussed that not everything online is true	88.7%	96.0%	90.6%
Discussed the potential dangers of the I	85.9%	97.2%	90.2%
Rules about the time the child can spend online	34.7%	42.6%	42.0%
Rules about the times of the day the child can be online	58.0%	70.7%	58.7%
Rules about the use of the I in the bedroom	47.1%	64.8%	59.6%
Rules about the pictures the child can post online	43.7%	64.3%	56.1%
Rules about the information the child can share	50.6%	64.9%	55.6%
Rules about with whom the child can chat	26.3%	49.2%	38.4%
Rules about who the child can add to the SN	35.5%	52.3%	40.7%
Being friends with a parent/with the child on the SN*	77.4%	70.3%	59.7%
Parents have access to the child's SN login credentials*	39.7%	52.8%	47.7%

SN = social network; I = internet; * not included in factor analyses
